# Characterisation of New Zealand Propolis from Different Regions Based on Its Volatile Organic Compounds

**DOI:** 10.3390/molecules29133143

**Published:** 2024-07-02

**Authors:** Ruby Mountford-McAuley, Alastair Robertson, Michelle Taylor, Andrea Clavijo McCormick

**Affiliations:** 1 School of Food Technology & Natural Sciences, Massey University, Palmerston North 4410, New Zealand; rubymountford8100km@gmail.com (R.M.-M.); a.w.robertson@massey.ac.nz (A.R.); 2 The New Zealand Institute for Plant and Food Research Limited, Hamilton 3214, New Zealand; michelle.taylor@plantandfood.co.nz; 3 School of Agriculture and Environment, Massey University, Palmerston North 4410, New Zealand

**Keywords:** biological activity, native plants, resin, poplar, terpenes, volatile organic compounds

## Abstract

Propolis is a bee product mainly consisting of plant resins and is used by bees to maintain the structural integrity of the colony. Propolis is known to contribute to bee health via its antimicrobial activity and is a valued product for human use owing to its nutritional and medicinal properties. Propolis is often characterised into seven categories depending on the resin source. New Zealand propolis is typically assumed as being poplar-type propolis, but few studies have chemically characterised New Zealand propolis to confirm or reject this assumption. Here, for the first time, we characterise propolis originating from different regions in New Zealand based on its volatile organic compounds, using gas chromatography coupled with mass spectrometry (GC-MS). To support this characterisation, we also collected and analysed resin samples from a variety of resin-producing plants (both native to New Zealand and introduced). Our findings suggest that bees mainly use poplar as a resin source, but also utilize native plant species to produce propolis. While regional variation did not allow for clear separation between samples, some patterns emerged, with samples from some regions having more chemical complexity and a higher contribution from native species (as suggested by a higher number of compounds unique to native species resin). Further studies are needed to accurately identify the botanical sources contributing to these samples. It may be also of interest to explore the biological activity of regional propolis samples and their potential nutritional or medicinal benefits.

## 1. Introduction

Propolis, is a blend derived from resin gathered by honey bees (*Apis mellifera*) and various other bee species from specific plants’ leaves, bark, and buds. Only a fraction of worker bees within the hive are designated to procure resin [1]. Following collection, bees engage in chewing the resin and combine it with salivary enzymes, beeswax, and traces of pollen to form propolis [2,3]. The bees employ propolis primarily for sealing unwanted crevices and upholding the hive’s structural integrity [4]; therefore, it is a crucial element in social immunity [5,6,7,8], exhibiting biological activity against pests and diseases [9,10,11].

Propolis, like other bee-derived substances, has been subject to thorough explorations for its medicinal potential. Among its array of health benefits, propolis exhibits antibacterial, antioxidative, antifungal, anti-inflammatory, anti-tumoral, and anti-carcinogenic properties [12,13,14,15,16,17]. Consequently, there is increasing interest in propolis and its utilization in dietary supplements and biocosmetics. This burgeoning interest has prompted efforts to investigate the botanical and regional origins of propolis worldwide, and their links to biological activity, e.g., [18,19,20,21,22,23,24].

Many factors affect the quality and yield of propolis. These include the botanical origin and availability of the resin, honey bee genetics, the health state of the colony, the hive structure, and the extraction and filtration processes used to obtain propolis extract [1,2,3,4,5,6,7,8,9,10,11,12,13,14,15,16,17,18]. Of these, the botanical source is essential to the biological activity of propolis, as most chemicals in the resin do not undergo chemical modification by bees [18,25,26].

Plants produce copious amounts of volatile organic compounds (VOCs) (i.e., scent or aroma compounds), which include secondary metabolites that serve ecological roles in plant pollination, defence, and competition, e.g., [27,28,29]. Volatile organic compounds are important contributors to the chemical signature of propolis and its pleasant aroma [30,31]. The appeal of this fragrance extends beyond human consumers: honey bees, too, are highly responsive to odours across various contexts [32]. Moreover, the volatile compounds found within propolis play a significant role in its biological efficacy against human and honey bee pathogens [33,34,35,36,37,38,39,40,41,42,43].

Plant volatiles are also excellent taxonomic indicators, providing unique chemical signatures that allow distinction between closely related plant species and varieties, e.g., [44,45,46]. Given that honey bees integrate plant resins into propolis with minimal chemical modification, the volatile elements within propolis serve as valuable tools for characterizing and identifying the botanical origins of propolis samples [47,48,49,50].

International lists often classify propolis into categories based on their geographical and botanical origin. Most studies identify seven propolis types: ‘Poplar’ derived from *Populus* spp. (poplar) found in Europe, northern and southern South America, and China; ‘Red’ derived from *Dalbergia ecastaphyllum* (coin vine) found in Cuba, Mexico, and Brazil; ‘Brazilian green’ derived from *Baccharis dracunculifolia* (alecrim) in Brazil; ‘Birch’ derived from *Betula* spp. (birch) found in Russia; ‘Mediterranean’ derived from conifers (e.g., pine) found in Greece, Sicily, Crete, and Malta; ‘Clusia’ derived from *Clusia* spp. found in Cuba and Venezuela; and ‘Pacific’, derived from a variety of native and endemic botanical sources (e.g., *Macaranga* spp.) and found in Taiwan, Indonesia, Pacific Islands, and parts of Japan [14,31,37]. When New Zealand propolis is listed, it is classified as European/temperate/poplar-type propolis, e.g., [37,38,39], but few studies have been conducted to confirm or reject this assumption.

The New Zealand honey bee product market is extensive. Of particular favour in the international market is the close association of native plant sources and bee products such as mānuka (*Leptospermum scoparium*) honey [45]. Despite this association and international demand, New Zealand propolis is often absent from global propolis lists. Very little is known about the chemical composition of New Zealand propolis. This work aims to fill this knowledge gap by characterizing the volatile compounds of native and poplar resin sources and comparing them with those present in New Zealand propolis produced by honey bee colonies during spring and autumn from various geographical regions.

In this study, resin samples were collected from common native and introduced (i.e., poplar) botanical sources in New Zealand during both autumn and spring. Volatile compounds were extracted using direct solvent extraction (in hexane 95%) and tentatively identified using gas chromatography–mass spectrometry (GC-MS). Propolis was collected and supplied by beekeepers around New Zealand in autumn and spring. Propolis samples were categorised based on their respective supply regions. Volatile compounds were extracted using headspace solid-phase microextraction (HS-SPME) and then tentatively identified using GC-MS. The volatile profiles of resin samples were compared with those of propolis samples to establish potential botanical origins for the propolis. This paper reports and expands on the findings of a postgraduate thesis conducted at Massey University, New Zealand [51]. This is the first study to test the widespread assumption of New Zealand propolis being poplar-type propolis and to investigate the potential contribution of native botanical sources to New Zealand propolis. It is also the most comprehensive study of regional variation in propolis conducted in New Zealand to date.

## 2. Results and Discussion

### 2.1. Resin Analyses

**Figure 1:**
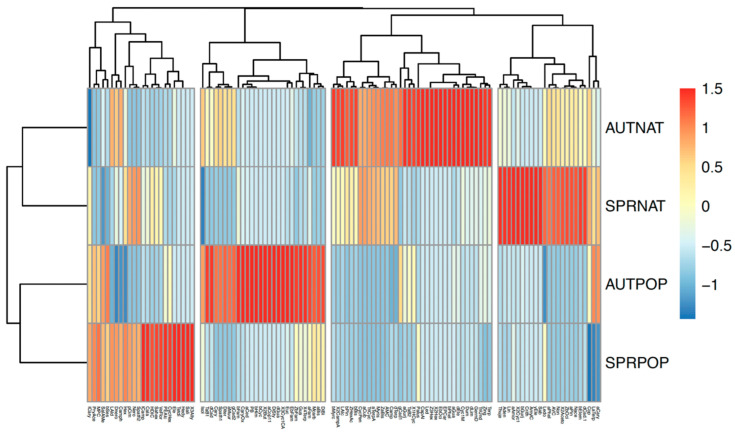
Separation of New Zealand resin samples by season of collection (AUT: autumn, SPR: spring) and botanical origin (NAT: native, POP: poplar) based on abundance of compounds. 1.5 indicates high abundance and −1.5 low abundance. At the bottom, the abbreviation of the compounds responsible for the separation is listed. For details on the compound abbreviations, see Supplementary Table S1.

A total of 111 compounds were tentatively identified from the 64 resin samples we analysed (for a list of compounds and their abbreviations, see Supplementary Table S1; for estimated abundances, see Supplementary Tables S2–S5). All samples were rich in terpenes (commonly found in resins). Different isomers of farnesene and pinene (among others) were found in both poplar and native samples. Autumn and spring poplar samples were characterised by the presence of methallyl carbinol (Mcarb) in varying amounts. Spring poplar samples were characterised by a higher abundance of compounds such as 3-allylguaiacol (X3Ally) and salicylal (Sali), while autumn samples consistently contained high amounts of eucalyptol (Euc) and caryophyllene (Cary), among others (Figure 1, Supplementary Tables S2 and S3). Native samples displayed very different profiles, reflecting their varied botanical origins. We identified multiple compounds in native samples that were absent in poplar samples, including dumasin (Dum), α-cubebene (aCub), crithmene (Crith), myristicin (Myris), β-bisabolene (bBis), and (*Z*)-α-bisabolene (ZaBis) (Figure 1, Supplementary Tables S4 and S5). Many of these were specific to just one native plant species.

**Figure 2:**
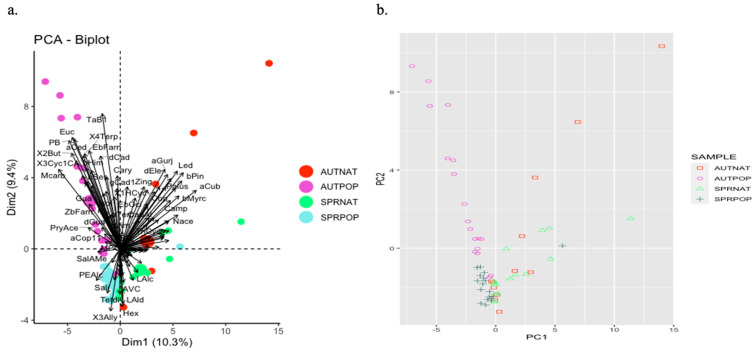
(**a**) Grouping of New Zealand resin samples collected in spring (SPR) and autumn (AUT) from poplar (POP) and native (NAT) plants along two dimensions, resulting from a dimension reduction method–principal component analysis (PCA). Each circle represents an individual sample. The larger circles are the means of each group of samples (separated by the combinations of source and season). Vector arrows with compound names represent which compounds contribute to the separation (the larger the vector, the more it contributes, and the direction of the arrow indicates to which component). For details on the compound abbreviations, see Supplementary Table S1. (**b**) Simplified version of the PCA showing the distribution of samples along the two principal components. The figure shows separation between poplar and native samples along the first component (PC1), and between autumn and spring samples along the second component (PC2).

A principal component analysis (PCA) showed some separation based on botanical source and season (Figure 2). The first principal component (PC1) explained 10.3% of the variability, where the samples were best separated based on their botanical origin (poplar vs. native). PC1 was characterised by high scores for compounds such as ledene (Led) and α-cubebene (aCub) (only present in native samples). Principal component two (PC2) accounted for 9.4% of the total variance and separated samples largely based on the season when they were collected. Compounds such as eucalyptol (Euc), (*E*)-α-bergamotene (TaB1), and camphene (Camp) (present in higher amounts in autumn samples) were the main contributors to this dimension.

### 2.2. Propolis Analyses

**Figure 3:**
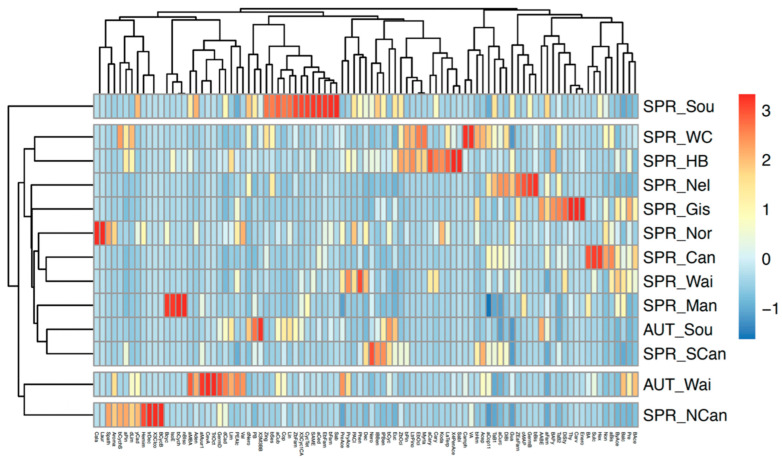
Separation of New Zealand propolis samples by season of collection (AUT: autumn, SPR: spring) and region (refer to Table 1 for details on region labels) based on relative abundance of compounds; 3 indicates a high abundance and −1.5 a low abundance. At the bottom, the abbreviation of the compounds responsible for the separation is listed. For details on the compound abbreviations, see Supplementary Table S1.

Ninety-one (91) compounds were tentatively identified in 73 propolis samples (Supplementary Tables S6–S9). While none of the compounds were present in all propolis samples, eleven compounds were consistently identified in all autumn samples, and up to forty compounds were detected in all spring samples, with some compounds being shared among samples and seasons. The two most common compounds were prenal acetate (PreAce) and (*E*)-α-bergamotene (TaB1), identified in samples from all regions and seasons. α-Copaen-11-ol (aCop11) was identified in all but one of the groups of propolis samples. Three compounds, benzyl alcohol (Balc), phenylethyl alcohol (PEAlc), and α-curcumene (aCurc), were identified in all but two of the groups of propolis samples. Similarly, α-pinene (aPin) and β-pinene (bPin) were consistently present in most of the samples. Many compounds were rare and identified in only one set of samples (Tables S5–S8, Figure 3).

**Figure 4:**
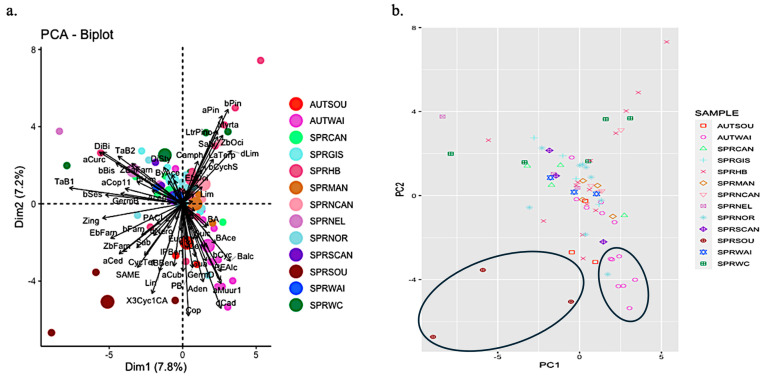
(**a**) Grouping of New Zealand propolis samples collected from different regions in spring (SPR) and autumn (AUT) along two dimensions, resulting from a dimension reduction method—principal component analysis (PCA). For details on the region codes see Table 1. Each circle represents an individual sample. The larger circles are the mean of each group of samples (separated by the combination of season and geographic origin). Vector arrows with compound names represent which compounds contribute to the separation (the larger the vector, the more it contributes, and the direction of the arrow indicates to which component). For details on the compound abbreviations, see Supplementary Table S1. (**b**) Simplified version of the PCA showing the sample distribution along two principal components, showing high overlap between samples and a few distinct clusters (inside black ellipses).

A PCA (Figure 4) revealed poor discrimination of the propolis samples based on their volatile components (7.8% in dimension 1 and 7.2% in dimension 2). There was no significant separation based on geographical location or season. However, some samples appear to form distinct clusters, e.g., those collected from Waikato in autumn (AUTWAI) and Southland in spring (SPRSOU).

Figure 4 illustrates a separation of seven of the Waikato samples collected in autumn, distinguished by the presence of compounds such as prenal acetate (PreAce), benzyl alcohol (Balc), phenylethyl alcohol (PEAlc), and α-copaen-11-ol (aCop11). However, no single compound was identified in all autumn Waikato samples, and those that were identified varied in their quantities.

In contrast, the cluster formed by the three spring Southland samples was characterised by α-cedrene (aCed), linalool (Lin), and 3-Cyclohexen-1-carboxaldehyde, 1,3,4-trimethyl- (3Cyc1CA), which were found only in a few other samples (see Supplementary Table S9). Even within this cluster, variation was evident, with compounds like prenyl acetate (PryAce), α-curcumene (aCurc), β-farnesene (bFarn), δ-cadinene (dCad), and guaiol (Gua) being detected in only two of the samples. Figure 4 highlights this divergence, particularly with one spring Southland sample distinctly separated from the others, lacking prenyl acetate (PryAce), benzyl alcohol (Balc), α-curcumene (aCurc), and β-pinene (bPin), which were identified in 39 and 29 out of 73 samples, respectively. Instead, this sample featured germacrene D (GermD) and sabinene (Sab), identified in only two other samples.

Another notable outlier was a spring sample from Hawke’s Bay (top right of Figure 4b), characterised by the absence of prenal acetate (PreAce), benzyl alcohol (Balc), and (*Z*)-α-bergamotene (TaB2), which were present in 51, 36, and 52 out of 73 samples, respectively. Conversely, this sample contained δ-limonene (dLim), myrtenol (Myrta), and sabinol (Sabi), identified in only five, two, and one other samples, respectively.

The results (Figure 3) also suggest distinct profiles from spring North Canterbury samples and support regional differences throughout; e.g., Manawatū-Whanganui samples contained β-Cyclocitral (Bcyc), Isoeugenol (IsoE), Bicyclo[2.2.1]heptan-3-one, 6,6-dimethyl-2-methylene-(bCych), and n-Butyl isobutyrate (nBiso), which were either absent or uncommon elsewhere.

### 2.3. Comparison of Resin and Propolis Samples

**Figure 5:**
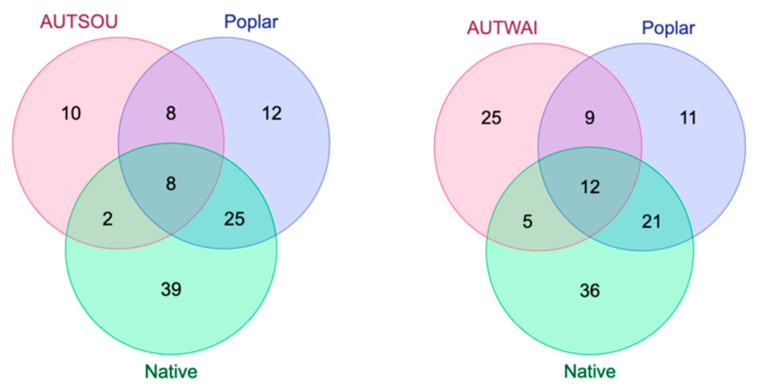
Comparison of compounds present in autumn propolis samples (pink circles) from two different New Zealand regions (SOU = Southland and WAI = Waikato), and resin samples collected during the same season from poplar (blue) and native plants (green).

**Figure 6:**
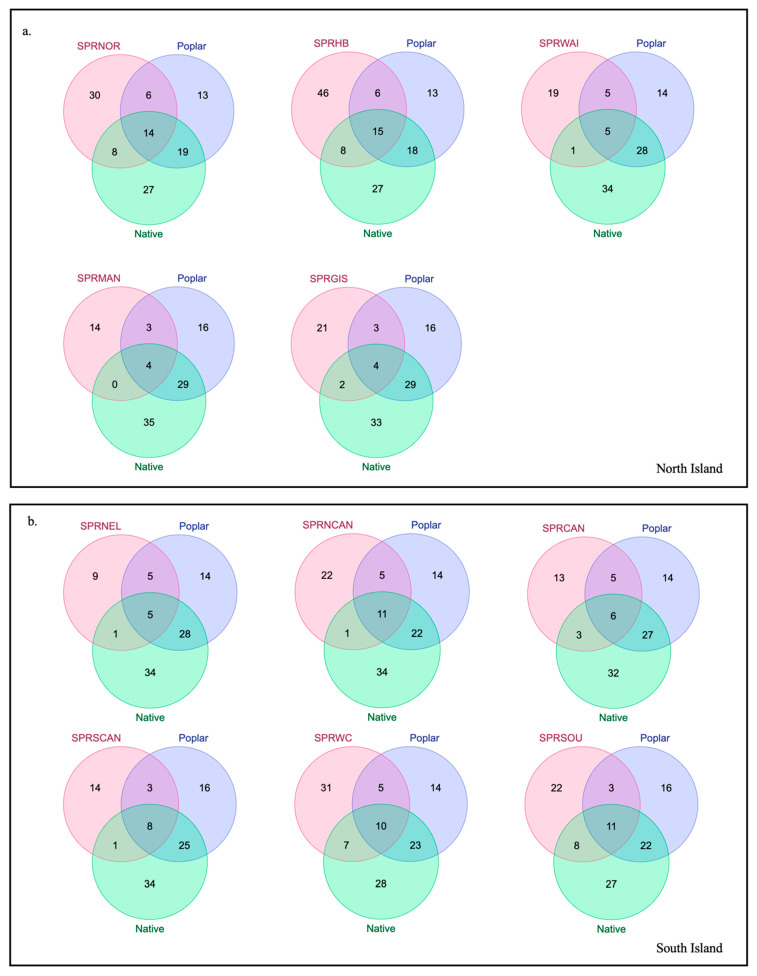
Comparison of compounds present in spring propolis samples (pink circles) from different regions in the North (**a**) and South (**b**) Islands of New Zealand, and resin samples collected during the same season from poplar (blue) and native plants (green). For region abbreviations refer to Table 1.

When comparing resin and propolis samples based on shared compounds, all propolis samples were found to contain compounds uniquely present in poplar resin (Figure 5 and Figure 6). For instance, α-copaen-11-ol (aCop11) was identified in 40 out of 72 propolis samples, as well as in one autumn Pakaraka (*Populus* × *euramericana* ‘Pakaraka’) resin sample and all three autumn Weraiti (*P.* × *euramericana* ‘Weraiti’) resin samples. Phenylethyl alcohol (PEAlc) was detected in 33 of the 72 propolis samples and in all autumn Argyle (*P.* × *euramericana* ‘Argyle’) and Weraiti resin samples, as well as in two autumn Selwyn (*P.* × *euramericana* ‘Selwyn’) resin samples and all spring poplar resin samples. β-Himachalene (bHim) was found in autumn Waikato propolis samples, as well as in spring propolis samples collected from Gisborne, Canterbury, West Coast, Hawke’s Bay, and Northland, and in all autumn Fraser (*P.* × *euramericana* ‘Fraser’), Pakaraka, Selwyn, and Weraiti resin samples.

However, except for one propolis sample, the majority also contained compounds unique to resin from native species (Figure 5 and Figure 6). For instance, nonanal (Non) was detected in spring propolis samples from Gisborne, Canterbury, West Coast, Southland, and Northland, as well as in autumn kawakawa (*Piper excelsum*) and spring rewarewa (*Knightia excelsa*) resin samples, but not in any poplar resin samples. The compound α-cubebene (aCub) was found in autumn propolis samples from Waikato and Southland, and spring propolis samples from Northland and Southland. It was also identified in autumn resin samples of kawakawa, kōhūhū (*Pittosporum tenuifolium*), and kauri (*Agathis australis*) and spring resin samples of tōtara (*Podocarpus totara*), mānuka (*Leptospermum scoparium*), kānuka (*Kunzea ericoides*), and kauri. Similarly, the compound β-bisabolene (bBis) was present in spring propolis samples from Nelson, West Coast, Hawke’s Bay, and Northland; in autumn resin samples of kawakawa, kōhūhū, and ngaio (*Myoporum laetum*); and in spring resin samples of kawakawa and ngaio. E-β-Ocimene (EbOci) was identified in spring propolis samples from West Coast and Hawke’s Bay and in autumn resin samples of lemonwood (*Pittosporum eugenioides*) and kauri. None of these compounds was found in polar resins. Some compounds found in the propolis samples were not detected in any of the sampled botanical species.

This comparison also reveals some regional differences in propolis samples (Figure 6). The samples from the upper North Island regions like Northland (SPRNOR) and Hawke’s Bay (SPRHB) had the highest number of compounds overall and more propolis compounds, which were found only in native resins (eight each). In the South Island, a similar pattern was true for Southland (SPRSOU) and the West Coast (SPRWC) regions. In contrast, the remaining samples had typically fewer compounds and only a minor contribution from compounds unique to resins from native species. Specifically, the Manawatū-Whanganui sample (SPRMAN) had no compounds unique to native species. The Nelson (SPRNEL) and Manawatū-Whanganui (SPRMAN) propolis samples had the lowest number of compounds overall (20 and 21, respectively).

### 2.4. Discussion

We characterised the volatiles of 64 resin samples, including both native and poplar representatives collected in spring and autumn, and tentatively characterised 73 propolis samples collected in spring and autumn across different regions of New Zealand. Resins showed good separation between samples collected in autumn and in spring and between poplar and native samples (Figure 1 and Figure 2). However, there was high variability and overlap in the propolis samples (Figure 3 and Figure 4). Nevertheless, some samples from particular provenances and seasons clustered away from others, showing distinct chemical profiles, for example, samples collected from Southland in spring and from Waikato in autumn.

When comparing resin and propolis samples based on shared compounds (Figure 5 and Figure 6), we found that all the propolis samples contained compounds unique to poplar resin, such as α-copaen-11-ol (aCop11) and phenylethyl alcohol (PEAlc), suggesting this was the main botanical source. This is in keeping with the assumption that New Zealand propolis is ‘poplar-type’. However, most of the propolis samples also contained at least one compound that was not detected in the poplar resin but present in the resin of native plants (e.g., nonanal and α-Cubebene). This evidence suggests that other plant species also contributed resin to most of the propolis samples. Some regions like Northland, Hawke’s Bay, and Southland showed more complex chemical profiles with a higher contribution of compounds that were also found in native resins, suggesting that they might have come from these plant sources. This may reflect a higher availability of native plant sources in the areas where the propolis was collected, in contrast to more agricultural regions such as Manawatū-Whanganui, Nelson, and Canterbury (where propolis samples had fewer compounds overall).

While poplar resins tended to cluster together, significant variability was observed among resin samples from closely related species and across different seasons. This variability among poplar species has also been noted in previous studies; for example, Drescher et al. [52] identified similar variations when comparing resin collected from different colonies of resin-foraging honey bees for propolis production. Among poplar, birch, horse chestnut, and coniferous resins, poplars exhibited the highest rates of intraspecific variability. Canadian poplar (*Poplar × canadensis*) resin, in particular, varied significantly between individuals from different locations and even among neighbouring trees, with distinct chemotypes characterised. Several propolis samples did not clearly match a single resin source. Instead, they contained numerous compounds not found in poplar resin. This suggests that while poplar resin probably constituted a major component of these samples, other resin sources must have also been involved.

In other studies, VOCs have been utilized to establish links between propolis and its botanical origin. For instance, Agüero et al. [53] compared Andean Argentinian propolis samples with exudates from the native *Larrea nitida* and found a close match, suggesting substantial evidence that the propolis studied originated from *L. nitida*. However, several compounds were exclusively detected in propolis samples, implying the involvement of additional botanical sources, as observed in our study.

Similar observations were made by Cheng et al. [54], who collected samples from four different regions of China. They identified nine volatile compounds common to all four samples, with an additional ten compounds present in three of the four samples. Despite some similarities across regions, several compounds were unique to specific samples, indicating regional differences. Principal component analysis (PCA) further revealed distinct clusters for samples from each region, underscoring the influence of geographical origin on variation.

We also found some patterns of regional variation that are likely to reflect the abundance of native species in the area, with propolis from agricultural regions having less chemical diversity. It would be interesting to further explore propolis samples from the regions with more diversity of native plants, to accurately assess their resin sources and investigate their biological activity and medicinal properties.

Brazil has been at the forefront of distinguishing propolis into chemotypes based on its botanical origins. Researchers have successfully categorised propolis types into red, green, and brown varieties, primarily determined by their geographical and botanical origins. For instance, Machado et al. [55] compared the flavonoid and phenolic composition of propolis samples from different regions, confirming these distinctions. Additionally, scientists have pinpointed the likely botanical sources of each propolis type: red propolis originates from *Dalbergia ecastaphyllum* [56], green propolis from *Baccharis dracunculifolia* [57], and brown propolis from species within the *Copaifera genus* [58].

Clear separation may be feasible when the botanical species are very unique to a specific region. However, this may be challenging in New Zealand, where most natives are widely distributed and often co-exist with introduced plants (like poplar). Nevertheless, it would be of interest to further explore the difference between Northland and Southland samples, which, despite being geographically distant, both show a high chemical diversity and potentially high contribution from native species.

In contrast to some studies, which have successfully grouped propolis types by region, Falcão et al. [48] found that volatile oils isolated from propolis samples collected throughout Portugal did not allow for regional grouping based on geographical origin. However, correlations were established between the chemical compositions of propolis samples and those of resin samples collected from nearby plants.

Several key volatile compounds identified in propolis samples from New Zealand included (Z)-α-bergamotene (TaB2), α-curcumene (aCurc), α-copaen-11-ol (aCop11), α-pinene (aPin), and β-pinene (bPin). These compounds are commonly found in various plant species and have been identified in different types of propolis, such as red [56] and green Brazilian propolis [57], and Cypriot propolis [58]. Owing to the widespread presence of these terpenoids in the plant kingdom, attributing them to a single plant species is challenging.

The monoterpenes α- and β-pinene are recognized for their diverse therapeutic properties. Salehi et al. [59] recently conducted a comprehensive review highlighting their fungicidal, antimicrobial, antibacterial, and antiviral properties. Moreover, both compounds have demonstrated inhibitory effects on leukaemia and breast cancer.

Similarly, α-curcumene has been identified as a significant bioactive compound with notable properties. For example, α-curcumene, along with other active components found in ginger (α-farnesene, β-sesquiphelladrene, and zingiberin), showed potential in inhibiting the activity of the COVID-19 virus [60].

α-Copaen-11-ol was identified in all the propolis groups apart from the spring Manawatū-Whanganui samples. α-Copaen-11-ol has been identified as one of the most important components of Hamadan propolis, which has demonstrated anti-cancer activity against HCT116 cells [61]. Bergamotenes (such as (Z)-α-bergamotene) are bicyclic sesquiterpenes; are found in plants, insects, and fungi; and have been shown to possess diverse biological activities such as antioxidant, anti-inflammatory, immunosuppressive, cytotoxic, antimicrobial, antidiabetic, and insecticidal effects [62]. More research is needed to explore the biological activity of the compounds contributed by native species, since these could improve the medicinal and nutritional properties of propolis.

Our research indicates that New Zealand native plants are utilized by bees as a source of resin for propolis production. Moreover, we identified over 60 compounds in the propolis samples that were absent in any of the resin samples. Possible explanations include (1) additional botanical species may be contributing to New Zealand propolis; (2) there might be chemical alterations of the original resin compounds during propolis production; or (3) these compounds could originate from sources unrelated to the botanical origin of the propolis. There is some supporting evidence for the involvement of other botanical sources in New Zealand propolis. For instance, α-curcumene, a compound known for its botanical origin and biological activity [55], was found in more than half our propolis samples, but was absent in all resin samples analysed. Additionally, limonene was present in eight propolis samples but not detected in any of our resin samples, despite its botanical origin and previous identification in Cypriot propolis [58]. Nonetheless, further investigations are necessary to provide a definitive explanation.

## 3. Materials and Methods

### 3.1. Resin Sample Collection and Extraction

In autumn, we collected a total of 31 leaf bud samples for resin analysis: 13 from native species and 18 from poplar hybrids. During spring, we gathered 34 resin samples, including 16 from native species and 18 from poplar hybrids (Table 2). Poplar resin samples were obtained from Plant and Food Research’s poplar and willow orchard in Aokautere, New Zealand, in both April and November 2021. Apical leaf buds were harvested from six *Populus* × *euramericana* hybrids, specifically ‘Veronese’, ‘Pakaraka’, ‘Argyle’, ‘Selwyn’, ‘Fraser’, and ‘Weraiti’ (all common in New Zealand), with three individuals per clone. Buds were promptly chilled on ice until extraction later that day. Resin samples from native New Zealand plants were collected in April 2021 from Massey University campus in Palmerston North, and in November 2021 from Otari Wilton’s Bush, a native botanic garden and forest reserve in Wilton. All samples were handled and stored under identical conditions to ensure consistency. Resin samples were collected directly from either native plants or poplar varieties in autumn and spring 2021. AUT indicates autumn samples and SPR indicates spring samples; the remainder of the ID relates to the plant species sampled.

For extraction, one gram (1 g) of leaf buds was weighed and placed into a conical flask. Each flask received 10 mL of 95% hexane and an internal standard (10 nanograms per microlitre of nonyl acetate), after which they were sealed with cling film. The samples were left to sit for five hours at 16 °C. Following this, the leaf buds were removed, and the flasks were resealed and stored overnight at −80 °C. Each sample was then filtered through a 0.2 μm mesh filter, and 200 μL of the filtrate was transferred into vials and stored at −80 °C until chemical analysis. Results are shown as estimated abundance in nanograms per gram of fresh weight (of leaf buds).

### 3.2. Propolis Sample Collection

**Figure 7:**
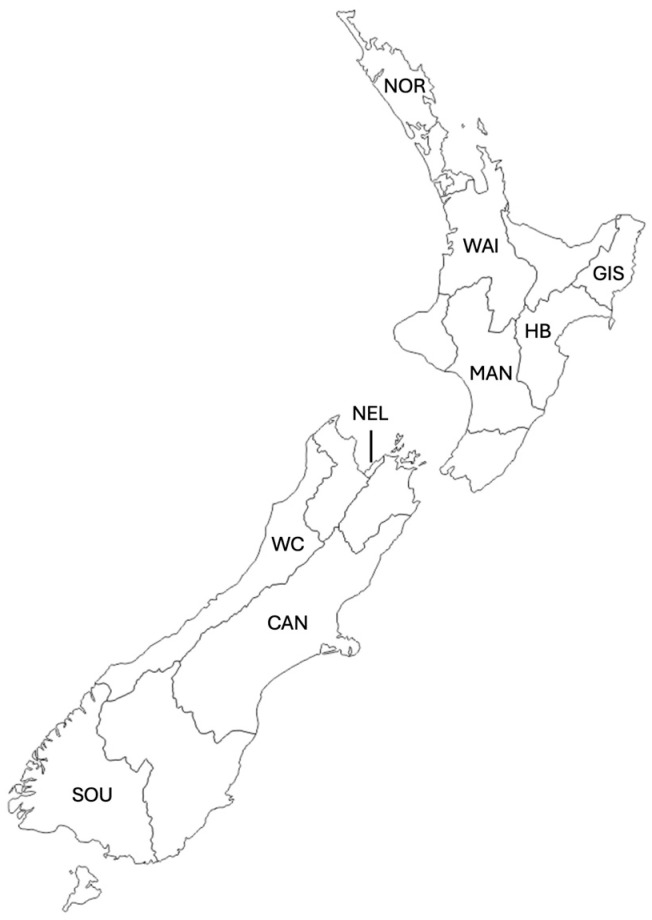
Map of the regions of New Zealand. Sampled regions (from top to bottom) are labelled as follows: Northland (NOR), Waikato (WAI), Gisborne (GIS), Hawke’s Bay (HB), Manawatū–Whanganui (MAN), Nelson (NEL), West Coast (WC), Canterbury (CAN), and Southland (SOU).

Nine regions were sampled: Northland, Waikato, Gisborne, Hawke’s Bay, Taranaki, Manawatū-Whanganui, Nelson, North Canterbury, Canterbury, South Canterbury, West Coast, and Southland (Table 1 and Figure 7). We collected a total of seventy-two (72) propolis samples, sixteen (16) in autumn and fifty-six (56) in spring.

In April 2021 (autumn), Arataki Honey Ltd collected propolis mats (plastic mats designed with grooves for propolis deposition and collection). These mats were grouped by the originating apiary, wrapped in plastic and burlap sacks, and then shipped to Massey University, Palmerston North. The propolis was extracted from the mats and stored at −4 °C until analysis.

During spring 2021, Beetek propolis mats (Ecrotek, Auckland, New Zealand) were distributed to beekeepers for placement on hives starting in late September. Beekeepers received between 5 and 25 mats, based on the size of their apiaries and operational area. The mats remained in the hives for a period ranging from five to nine weeks. After collection from each site, the mats were wrapped in tinfoil, placed in sealed plastic bags, and transported to Massey University on ice. The propolis extracted from these mats was similarly stored at −4 °C until analysis.

Two propolis samples collected in autumn, AUT8 and AUT12, were excluded from further processing owing to sample degradation. The number of replicates per region/season varied because the mats were provided by beekeepers. Information on the flora within three kilometres of the hives was requested from beekeepers but was found to be vague (mostly common or regional plant names), leading to its exclusion from subsequent analyses.

### 3.3. Headspace Solid-Phase Microextraction (HS-SPME) Procedure for Propolis Samples

We employed a modified approach based on the headspace solid-phase microextraction–gas chromatography–mass spectrometry (HS-SPME-GC-MS) methods outlined in [50] for analysing propolis samples. Extraction was conducted using a manual holder and a 100-μm polydimethylsiloxane (PDMS) fibre (Supelco, Bellefonte, PA, USA). Before gas chromatography–mass spectrometry (GC-MS) analysis, the fibres were conditioned in the GC injector following the manufacturer’s instructions. Two grams (2 g) of the sample (either resin or propolis) was placed in a 10-mL round-bottom headspace vial, which was sealed with a magnetic screw cap featuring a Silicone/PTFE septum. The sample was heated in a thermostatic bath at 75 °C for 30 min. The solid-phase microextraction (SPME) device was then inserted into the vial, and the fibre was exposed to the headspace for 15 min. The samples were analysed using direct injection GC-MS. Following analysis by GC-MS, the SPME fibre was reconditioned for five minutes in the GC injector port at 250 °C before reuse. Sample extraction and injection were conducted under highly controlled conditions to allow for comparability among samples.

### 3.4. Gas Chromatography–Mass Spectrometry Analyses for Resin and Propolis Samples

The compounds were chromatographically separated using a 30 m × 250 µm × 0.25 µm TG-5MS capillary column. Helium served as the carrier gas at a pressure of 53.5 kPa, with a linear velocity of 36.6 cm/s, total flow rate of 14.0 mL/min, and purge flow of 3.0 mL/min. The initial oven temperature was set at 50°C and held for three minutes, followed by an increase of 9 °C per minute until reaching 200 °C, which was maintained for three minutes. Identification of compounds was tentative, achieved by comparing target spectra with the mass spectra library from the National Institute of Standards and Technology (NIST) using GC-MS post-run analysis software (GCMS-QP2020 Series) provided by the instrument manufacturer (Shimadzu Corporation, Kyoto, Japan). Compound identities were considered acceptable at 80% certainty. Resin samples peaks were quantified relative to an internal standard, then normalized by the dry weight of the leaf buds and sampling duration (in hours) to estimate emissions per dry weight per hour. We ran control samples using identical procedures as described above, without resin or propolis, to identify and exclude any contaminants found in these samples from further analyses. For propolis samples, quantification was not feasible due to the method used; therefore, we used the area under the peak to estimate relative abundance.

### 3.5. Statistical Analyses

We conducted statistical analyses using R Studio version 2021.09.1 (Integrated Development Environment for R; RStudio, PBC, Boston, MA, USA). To explore patterns in the dataset related to season and geographical origin, we performed principal component analysis (PCA) using the R packages “prcomp”, “FactoMineR”, and heatmaps using “pheatmap”. This allowed us to visualize potential clustering and separation of samples based on these variables.

## 4. Conclusions

This study represents the first effort to test the widespread assumption that New Zealand propolis is poplar-type and explore its regional variation. To this end, we chemically characterised and compared propolis samples from different regions and resins from common poplar hybrids and native plant species. While all propolis samples contained compounds unique to poplar resin, nearly all samples (with one exception) also included compounds exclusive to native resins. Additionally, certain compounds of known botanical origin were present in propolis but absent from tested resin samples, suggesting that additional botanical sources not included in this study also contribute to New Zealand propolis. These findings indicate that while poplar resin probably plays a primary role in New Zealand propolis composition, bees also utilize resin from native plant species. Although the geographical origin of propolis samples did not definitively explain all variations observed, some regional patterns indicative of native plant presence and abundance were identified. The outcomes of this research lay a robust foundation for further exploration into geographic variations in New Zealand propolis, the contribution of various botanical sources, and the potential for refining the classification of New Zealand propolis. Investigating the biological activity of different New Zealand propolis, including their nutritional and medicinal properties is also a promising avenue for future research.

## Figures and Tables

**Table 1:** Labelling key for New Zealand propolis samples, with AUT indicating autumn samples and SPR indicating spring samples. For the Canterbury region, we separated samples in North, Central, and South Canterbury.

Sample ID	Region	Number of Replicates
AUTWAI	Waikato	13
AUTSOU	Southland	3
SPRNOR	Northland	9
SPRWAI	Waikato	3
SPRGIS	Gisborne	4
SPRHB	Hawke’s Bay	14
SPRMAN	Manawatū-Whanganui	4
SPRNEL	Nelson	3
SPRWC	West Coast	5
SPRNCAN	North Canterbury	4
SPRCAN	Central Canterbury	4
SPRSCAN	South Canterbury	4
SPRSOU	Southland	3

**Table 2:** Resin samples collected directly from either New Zealand native plants or poplar varieties in autumn and spring 2021. AUT indicates autumn samples and SPR indicates spring samples; the remainder of the ID relates to the plant species sampled.

Sample ID	Common Name of Plant	Scientific Name of Plant *
*Introduced*		
AUTA1-3	Argyle	*Populus* × *euramericana* ‘Argyle’
AUTF1-3	Fraser	*Populus* × *euramericana* ‘Fraser’
AUTP1-3	Pakaraka	*Populus* × *euramericana* ‘Pakaraka’
AUTS1-3	Selwyn	*Populus* × *euramericana* ‘Selwyn’
AUTV1-3	Veronese	*Populus* × *euramericana* ‘Veronese’
AUTW1-3	Weraiti	*Populus* × *euramericana* ‘Weraiti’
*Native*		
AUTC1	Pūriri	*Vitex lucens*
AUTG1	Karaka	*Corynocarpus laevigatus*
AUTH1-2	Whau	*Entelea arborescens*
AUTI1	Pukatea	*Laurelia novae-zelandiae*
AUTK1-2	Kawakawa	*Piper excelsum*
AUTL1	Lemonwood	*Pittosporum eugenioides*
AUTN1	Kānuka	*Kunzea ericoides*
AUTO1	Kōhūhū	*Pittosporum tenuifolium*
AUTQ1	Kauri	*Agathis australis*
AUTR1	Ngaio	*Myoporum laetum*
AUTT1	Māpou	*Myrsine australis*
*Introduced*		
SPRA1-3	‘Argyle’	*Populus* × *euramericana* ‘Argyle’
SPRF1-3	‘Fraser’	*Populus* × *euramericana* ‘Fraser’
SPRP1-3	‘Pakaraka’	*Populus* × *euramericana* ‘Pakaraka’
SPRS1-3	‘Selwyn’	*Populus* × *euramericana* ‘Selwyn’
SPRV1-3	‘Veronese’	*Populus* × *euramericana* ‘Veronese’
SPRW1-3	‘Weraiti’	*Populus* × *euramericana* ‘Weraiti’
*Native*		
SPRE1	Tōtara	*Podocarpus totara*
SPRD1	Rewarewa	*Knightia excelsa*
SPRH1	Whau	*Entelea arborescens*
SPRK1	Kawakawa	*Piper excelsum*
SPRM1	Māhoe	*Melicytus ramiflorus*
SPRI1	Pukatea	*Laurelia novae-zelandiae*
SPRJ1	Mānuka	*Leptospermum scoparium*
SPRN1	Kānuka	*Kunzea ericoides*
SPRO1	Kōhūhū	*Pittosporum tenuifolium*
SPRQ1	Kauri	*Agathis australis*
SPRR1	Ngaio	*Myoporum laetum*
SPRT1	Māpou	*Myrsine australis*
SPRU1	Tī Kōuka	*Cordyline australis*
SPRX1	Harakeke	*Phormium tenax*
SPRC1	Pūriri	*Vitex lucens*
SPRZ1	Kōwhai	*Sophora microphylla*

* All poplar hybrids sampled are collectively known as *Populus* × *euramericana*, therefore the common names of specific hybrids are added after the scientific name inside quotation marks.

## Data Availability

The datasets of this study are available in the Supplementary Materials.

## Supplementary Materials

The following supporting information can be downloaded at: https://www.mdpi.com/article/10.3390/molecules29133143/s1, Table S1: List of abbreviations used throughout the text and the corresponding compound names; Table S2: Tentative identification and estimated abundance (ng/g FW leaf bud) of compounds in poplar resin samples collected in autumn; Table S3: Tentative identification and estimated abundance (ng/g FW leaf bud) of compounds in poplar resin samples collected in spring; Table S4: Tentative identification and estimated abundance (ng/g FW leaf bud) of compounds in native resin samples collected in autumn; Table S5: Tentative identification and estimated abundance (ng/g FW leaf bud) of compounds in native resin samples collected in spring; Table S6: Tentative identification and area under the peak for compounds propolis samples collected in autumn; Table S7: Tentative identification and area under the peak for compounds propolis samples collected in spring in the upper half of the North Island of New Zealand, Table S8: Tentative identification and area under the peak for compounds propolis samples collected in spring from the lower half of the North Island of New Zealand; Table S9: Tentative identification and area under the peak for compounds propolis samples collected in spring from the South Island of New Zealand.
