# Supplementary material for: Characterisation of New Zealand Propolis from Different Regions Based on Its Volatile Organic Compounds
Source: Molecules. 2024 Jul 2;29(13):3143. doi: 10.3390/molecules29133143 (PMC11243487; doi:10.3390/molecules29133143)
Supplement: Supplementary file 1 [file molecules-29-03143-s001.zip › molecules-3076631-supplementary.pdf]

molecules

Characterisation of New Zealand Propolis from Different Regions Based on Its Volatile Organic Compounds

Ruby Mountford-McAuley [1], Alastair Robertson [1], Michelle Taylor [2] and Andrea Clavijo McCormick [3]*

[1] School of Food Technology & Natural Sciences, Massey University, Palmerston North 4410, New Zealand; rubymountford8100km@gmail.com (R.M.-M.); a.w.robertson@massey.ac.nz (A.R.)
[2] The New Zealand Institute for Plant and Food Research Limited, Hamilton 3214, New Zealand; michelle.taylor@plantandfood.co.nz (M.T.)
[3] School of Agriculture and Environment, Massey University, Palmerston North 4410, New Zealand
* Correspondence: a.c.mccormick@massey.ac.nz (A.C.M.)

Table S1. List of abbreviations used throughout the text and the corresponding compound names. The abbreviations are listed in alphabetical order.

Compound Abbreviation	Compound Name
1HCyc	1H-Cyclopental[1,3]cyclopropal[1,2]benzene, octahydro-7-methyl-3-methylene-4-(1-methylethyl)-, [3aS-3a α.,3b. β.,4. β.,7]
2Aceto	2-Acetoxydodecane
2But	2-Buten-1-ol, 2-methyl-
2CampA	2-Camphanol acetate
2Cyc1	2-Cyclopenten-1-one
2Hex	2-Hexanol
2M1Oc	2-Methyl-1-octanol
2Med	2-Methyldecalin
3Car	3-Carene
3CIco	3-Cyclohexyleicosane
3Cyc1CA	3-Cyclohexene-1-carboxaldehyde, 1,3,4-trimethyl-
3M3BB	3-Methyl-3-butenyl benzoate
4PenAce	4-Pentenyl acetate
4Terp	4-Terpineol
5Az	5-Azulenemethanol, 1,2,3,4,5,6,7,8-octahydro-.alpha.,.alpha.,3,8-tetramethyl-
7Endo	7-Endo-ethenyl-bicyclo[4,2,0]-oct-1-ene
7NBB	7-Norbornadienyl benzoate
AABE	Acrylic acid butyl ester
aAmor	α-Amorphene
aBis	α-Bisabolol
aCamp	α-Campholenal
aCary	α-Caryophyllene
aCed	α-Cedrene
Acep	Acetophenone
aCop11	α-Copaen-11-ol
aCub	α-Cubebene

aCurc	α-Curcumene
Aden	Alloaromadendrene
aFarn	α-Farnesene
aGua	α-Guiaene
aGurj	α-Gurjunene
aMBA	α-Methylbutyric acid
AMC	Amyl methyl carbinol
aMuur, aMuur1, aMuur2	α-Muurolene
AmylC	Amylcarbinol
Anis	Anisole
aPhel	α-Phellandrene
aPin	α-Pinene
Aroma	Aromadendrene
aTerpA	α-Terpenyl acetate
AVC	Amyl vinyl carbinol
b-Pino	β-Pinone
BA	Benzaldehyde
BAce	Benzyl acetate
Balc	Benzyl alcohol
BAPy	Benzoic acid 5-methyl-2-phenyl-2H-pyrazol-3-yl ester
bBis	β-Bisabolene
BCA	Benzenecarboxylic acid
bCyc	β-Cyclocitral
BCycB	Butylcyclohexane
BCycBu	Benzoylcyclobutane
bCych	Bicyclo[2.2.1]heptan-3-one, 6,6-dimethyl-2-methylene-
bElem	β-Elemene
bFarn	β-Farnesene
bGurj	β-Gurjene
bHim	β-Himachalene
bMyrc	β-Myrcene
bPhel	β-Phellandrene
bPin	β-Pinene
bSes	β-Sesquiphellandrene
ByAce	Benzyl acetone
Cala	Calamenene
Camp	Camphene
Camph	Camphor
CapA	Capric acid methyl ester
CapAl	Capric alcohol
Cary	Caryophyllene
CaryOx	Caryophyllene oxide
CevA	Cevadic acid
Cop	Copaene
Crith	Crithmene
Cume	Cumene
Cyc1M	Cyclohexene, 1-methyl-5-(1-methylethenyl)-
Cychept	Cycloheptane, 4-methylene-1-methyl-2-(2-methyl-1-propen-1-yl)-1-vinyl-
CycHex	Cyclohexene, 3,4-diethenyl-3-methyl-

CychexP	Cyclohexane, pentyl-
CycPen	Cyclopentanol
Cycpro	Cyclopropane, 1,1-dimethyl-2-(3-methyl-1,3-butadienyl)-
dCad	δ-Cadinene
dCado	δ-Cadinol
Dec	Decanal
dEle	δ-EIemene
dGua	δ-Guaiene
DiBi	6,6-Dimethyl-2-(3-oxobutyl)bicyclo[3.1.1]heptan-3-one
DiSty	3,4-Dimethoxystyrene
Diter	Di-tert-butylphenol
dLim	δ-Limonene
Dnero, dNero	δ-Nerolidol
dTerp	β-Terpineol
Dum	Dumasin
EbFarn	(*E*)-β-Farnesene
EbOci	(*E*)-β-Ocimene
Ele	Elemol
Enero	(*E*)-Nerolidol
EPCar	Ethylpropylcarbinol
Epig	Epiglobulol
Euc	Eucalyptol
gCad1, gCad2, yCad	γ-Cadinene
GermD	Germacrene D
Gua	Guaiol
HDIC	1-Hydroxy-1,7-dimethyl-4-isopropyl-2,7-cyclodecadiene
Hedy	Hedycaryol
Hemim	Hemimellitene
Hex	Hexanal
Hthy	Hexahydrothymol
IBBen	Isobutyl benzoate
ICary	Isocaryophyllene
IPBen	Isopentyl benzoate
IsoA	Isobutyric acid
IsoE	Isoeugenol
Isol	Isoledene
LAlc	Leaf alcohol
LAld	Leaf aldehyde
LaTerp	L-α-Terpineol
Laur	Lauric acid
lCamp	L-camphor
Led	Ledene
Ledo	Ledol
Lin	Linalool
LtrPino	L-(*E*)-Pinocarveol
Mcarb	Methallyl carbinol
MPC	Methyl propenyl carbinol
Myris	Myristicin
Myrta	Myrtenol

Myrto	Myrtenal
Nace	Neryl acetate
nBIso	n-Butyl isobutyrate
Nero	Nerolidol
Non	Nonanal
Nopi	(+)-Nopinone
oCy	o-cymene
oMAP	Ortho-Methoxyacetophenone
PA2Met	Propanoic acid, 2-methyl-, 1-(1,1-dimethylethyl)-2-methyl-1,3-propanediyl ester
PACI	Pentanoic acid, 2,2,4-trimethyl-3-carboxyisopropyl, isobutyl ester
Palus	Palustrol
PB	Prenyl benzoate
pCim	P-Cimene
PEAlc	Phenylethyl alcohol
Phen	Phenoprene
PinoC	Pinocarvone
PPr	Phenylethyl propionate
Pre	Prenal
PreAce	Prenal acetate
PryAce	Prenyl acetate
Sab	Sabinene
Sabi	Sabinol
Sahex	Sulfurous acid, hexyl octyl ester
SalAMe, SAME	Salicylic acid, methyl ester
Sali	Salicylal
Sati	(+)-Sativen
ScisV	(S)-cis-Verbenol
Spath, Spath1	Spathulenol
Sulc	Sulcatone
TaB1	(*E*)-α-Bergamotene
TaB2	(*Z*)-α-Bergamotene
Terp	Terpineol
tertB	tert-Butylbenzene
TetCyc	1,4,7,10-Tetraoxacyclododecan-2-one
Tetdi	Tetrahydrocyclopenta[1,3]dioxin-4-one
Thuje	Thujene
Thy	Thymol
TME	Thymol methyl ether
tr3Pin	(*Z*)-3-Pinanone
tr4Car	(*Z*)-4-caranone
trDec	(*Z*)-Decalin
TriCyc	Tricyclene
TriOct	Tricyclo[3.2.1.02,7]oct-3-ene, 2,3,4,5-tetramethyl-
TriUnd	Trilo[5.4.0.0(2,8)]undec-9-ene, 2,6,6,9-tetramethyl-
trPino	(Z)-Pinocarveol
VA	Valeric acid
Val	Valencene
VeFlor	PPVeridiflorol
Wid	Widdrol

X3Ally	3-Allylguaiacol
yEle	γ-Elemene
Z3Hex	(*Z*)-3-Hexenal
ZaBis	(*Z*)-α-Bisabolene
ZbFarn	(*Z*)-β-Farnesene
ZbOci	(*Z*)-β-Ocimene
ZHexAc	(*Z*)-3-Hexen-1-ol, acetate
Zing	Zingiberene

Table S2. Tentative identification and estimated abundance (ng/g FW leaf bud) of compounds in New Zealand poplar resin samples collected in autumn.

Compound Abbreviation	AUT A1	AUT A2	AUT A3	AUT F1	AUT F2	AUT F3	AUT P1	AUT P2	AUT P3	AUT S1	AUT S2	AUT S3	AUT V1	AUT V2	AUT V3	AUT W1	AUT W2	AUT W3
ZbFarn					79.77				21.04	210.35								
1HCyc											16.17							
2But	26.20	16.83	13.97	33.07	16.90		32.45		22.89	19.81		26.31			22.25	26.41	17.01	24.02
3Cyc1CA				14.35	6.26		14.37		5.50	11.79	7.77							
DiSty						14.70						19.38						
4Terp	6.40			5.62		5.37	6.14	4.31		6.08		11.23						
DiBi										140.48	105.07	150.84						
Aden		20.46					29.63	10.60	5.09							18.23	11.12	18.31
aCop11									20.85							48.53	28.09	43.56
aFarn										150.31	81.66	165.77						
aGua	29.57		14.08												12.03			
aGurj							44.25											
aMuur							84.01											
aPin	9.87	7.18	5.92	12.51		12.06	13.02	7.47	10.53	17.49	12.95	16.38						
aBis				42.80		27.97				17.29	21.13							
aCary	62.24	29.81	26.37	39.13	9.80	21.83				34.26	22.28	31.37				30.98	25.21	21.01
bGurj										22.16								
bPin	8.98			9.74			8.53			9.47		10.79						
Cary	310.26			43.68	13.91	22.84	47.22	21.06	26.66	17.23	16.82	19.69	10.90	9.92	22.05	138.63	86.95	123.55
CaryOx	56.06	38.05	29.68	10.73		5.73	15.93	7.07	5.13	8.01	7.64	11.57				28.08	26.83	27.47
Cop							23.32	17.29	6.49	11.14	46.87	12.09				10.96	3.05	7.26
CycHex							13.54											
dCad							227.40	66.41	46.03	91.98	73.77	75.84				55.75	27.56	28.53
dGua	67.99	23.15	28.42										8.82	13.06	26.47			
Diter		22.80												15.14	16.48			

Compound Abbreviation	AUT A1	AUT A2	AUT A3	AUT F1	AUT F2	AUT F3	AUT P1	AUT P2	AUT P3	AUT S1	AUT S2	AUT S3	AUT V1	AUT V2	AUT V3	AUT W1	AUT W2	AUT W3
Ele	34.68	7.13	14.38				17.49									45.48	20.80	24.17
Euc	71.47	46.34	40.17	99.29	21.69	52.96	75.94	40.32	44.61	94.35	59.86	102.33	11.53	9.11	21.92			
gCad1							169.69	37.81	25.60							37.31	17.17	23.67
gCad2												40.67						
Gua	1011.95	478.94	417.84	668.55	148.28	387.45							120.61	99.77	337.07			
ICary		176.76	161.86	60.42	21.33	34.61												
Isol										57.26								
LaTerp	12.41					5.77	5.76		7.18		6.28							
Lin	11.89			7.66		5.32												
Mcarb	42.03	23.05	28.66	29.26	8.11	18.38	57.93	23.23	37.96	58.31	49.02	80.74	9.06	11.23	38.12	37.00	25.20	31.21
MPC											15.13							
PEAlc	20.34	15.29	12.64								12.93	22.93				18.61	10.66	15.69
PryAce	13.56	17.42	36.14							12.91	9.33	31.07						
PB	73.36			31.89		19.54	56.56	19.10	46.70	71.81	64.70	56.08			23.43	38.37	29.50	39.73
SalAMe	16.17	17.30		16.44		10.49										25.16	15.32	26.33
Spath1	44.02											20.82						
Terp				10.18				2.39		13.09		16.85						
TaB1				157.43	41.54	88.08	74.19	16.17	56.37	80.78	60.87	85.85				13.82	9.55	19.64
TaB2				14.38			12.68	8.65	17.39			9.75						8.71
TriUnd						7.95												
Zing				76.13	16.62	8.96	11.43		6.73	38.07	27.57	39.45						
aCed				331.88	78.49	195.96	91.42	22.75	41.05	199.87	113.62	210.05						
bCyc	6.13					6.81						15.12				7.20	6.52	4.33
bFarn				363.13														
EbFarn				363.13		194.65	36.63	10.71			140.64	233.27						
bHim				152.63	35.93	76.57	563.54	124.89	349.07	113.41	46.18	98.08				99.67	71.81	118.3

Compound Abbreviation	AUT A1	AUT A2	AUT A3	AUT F1	AUT F2	AUT F3	AUT P1	AUT P2	AUT P3	AUT S1	AUT S2	AUT S3	AUT V1	AUT V2	AUT V3	AUT W1	AUT W2	AUT W3
bSes				121.52	40.50	67.60			23.66	62.78	47.23	33.69						
dEle				9.44														

Table S3. Tentative identification and estimated abundance (ng/g FW leaf bud) of compounds in New Zealand poplar resin samples collected in spring.

Compound Abbreviation	SPR A1	SPR A2	SPR A3	SPR F1	SPR F2	SPR F3	SPR P1	SPR P2	SPR P3	SPR S1	SPR S2	SPR S3	SPR V1	SPR V2	SPR V3	SPR W1	SPR W2	SPR W3
bMyrc	19.33																	
ZbFarn				25.18	23.85	29.24				18.00	23.12	21.51						
HDIC							18.91	11.69	20.19	14.40	16.49	19.02						
2CampA	7.26																	
X3Ally	214.93	418.07	343.76	190.98	173.21	209.43	292.12	320.06	363.37	104.09	125.80	120.91	375.32	341.35	361.61	370.27	365.13	387.2
4Terp	9.75																	
DiBi				34.35	31.99	41.93				23.31	27.75	24.07						
Aden	17.60																	
aAmor							4.88				24.67							
aFarn										25.52	31.74	33.64	47.51	37.07				
aGua	29.21																	
aPin	176.79	3.50																
aBis					12.70	15.22					16.80	11.05						
aCary	8.79	10.00	6.23															
bPin	67.52																	
Cala	215.52																	
Camp	60.51	5.00																
Camph	7.93	4.52																
Cop	38.06																	
CycHex							11.94	14.63	11.69									
dLim	27.10																	
Dnero	59.99			20.67		21.98												
dCad							28.53	19.92	22.37		9.33							
Ele	403.31	19.61	653.39	7.50		13.75	226.00	186.15	218.39				36.70	12.81	10.92	9.06	15.34	20.12
Euc	37.85	13.49	8.24				12.21	22.64	15.93		5.37							

Compound Abbreviation	SPR A1	SPR A2	SPR A3	SPR F1	SPR F2	SPR F3	SPR P1	SPR P2	SPR P3	SPR S1	SPR S2	SPR S3	SPR V1	SPR V2	SPR V3	SPR W1	SPR W2	SPR W3
GermD								19.13		20.81								
Gua		102.05	60.97	83.50	85.68	122.81							159.43	119.66	123.53			
Hedy																323.54	346.23	507.7
Hex	7.50	10.61	15.95				10.98		9.12				14.85	11.99	13.53		5.43	10.57
ICary	82.24	64.83	45.03	10.53		18.69	34.46	35.48	34.40	11.87	22.18	12.45	36.86	28.88	18.35	23.88	29.05	28.91
Isol							29.76											
lCamp	199.15																	
LAld	18.84	24.18	32.81				21.10	12.81	15.49	9.38			40.90	33.85	29.24	12.58		9.98
Ledo	40.04																	
Mcarb	7.59	3.00	9.74	18.20	20.44	18.99	35.67	52.77	43.35	17.92	28.12	17.29	9.55	8.94	6.93	13.62	20.35	16.35
MPC							20.99											
Nero				12.61														
pCim	23.45																	
Palus	14.25																	
PEAlc	21.77	25.45	38.17	17.39	17.23	24.66	46.71	53.06	67.79	21.93	19.45	18.75	10.98	15.20	10.49	26.26	56.44	22.37
PryAce				8.67		19.87		34.80	34.90	16.47	12.29	9.23					7.85	
Sali	249.63	679.82	602.46	990.47	2155.8	1269.4	423.37	235.94	599.06	687.79	832.62	712.28	254.83	187.15	168.93	591.08	1109.1	473.1
SalAMe						15.18										15.57	48.74	38.38
Spath2	94.92																	
Sahex										13.13	4.22		5.10					
Tetdi	283.03	45.69	0.00	180.18	79.90	57.04	107.28	137.59	108.49	68.55	101.81	100.36	86.75		141.63	140.04	148.29	154.7
TaB1				12.82	12.89					11.53	19.30	10.64						
TaB2						19.93												
VeFlor	258.67																	
Zing						9.03												
bSes				58.98	56.63	69.07				35.54	47.70	38.82						

Compound Abbreviation	SPR A1	SPR A2	SPR A3	SPR F1	SPR F2	SPR F3	SPR P1	SPR P2	SPR P3	SPR S1	SPR S2	SPR S3	SPR V1	SPR V2	SPR V3	SPR W1	SPR W2	SPR W3
dTerp	16.25																	
dCad									26.89									

Table S4. Tentative identification and estimated abundance (ng/g FW leaf bud) of compounds in New Zealand native resin samples collected in autumn.

Compound Abbreviation	AUTB1	AUTC1	AUTG1	AUTH1	AUTI1	AUTK1	AUTL1	AUTN1	AUTO1	AUTQ1	AUTR1	AUTT1
aCub						10.38			50.45	25.34		
aPhel										9.57		
bMyrc						6.92	19.59		100.90	16.31		77.58
bPhel							181.79		281.93	10.61		
ZbFarn						28.63						17.84
1HCyc										27.17		
2Aceto						6.43						
2CampA									62.19	17.91		
2Cyc1					32.58							
2Hex		15.21							10.25			
ZHexAc					13.29							
Z3Hex								133.31			140.46	19.06
Aden						34.97	37.02		180.95			
aGua							128.59		94.58			
aGurj									121.16			
aMuur										36.71		
aPin		387.35				22.01	27.17	53.67	1264.44	376.08		280.10
aCary									59.19			40.47
AMC						17.09						
AVC		7.61									156.15	12.50
bPin		23.72					45.66		121.46	76.79		
Camp						36.44			63.86	154.98		
Camph						8.34						
CapAl							14.59		4.66			
Cary						15.28	284.12		74.39	56.23		

Compound Abbreviation	AUTB1	AUTC1	AUTG1	AUTH1	AUTI1	AUTK1	AUTL1	AUTN1	AUTO1	AUTQ1	AUTR1	AUTT1
CaryOx							18.22					
Cop		17.04					17.39		6.67	78.07		
Cyc1M										56.56		54.78
CycPen					13.01							
dLim		20.01					673.20		20.94	42.20		
Dnero		8.93										34.82
dCad		12.86								154.15		
Diter			12.72	9.65								
Dum					157.27							
Ele							246.43		14.14			
EPCar		14.77			9.32							9.05
Euc						59.81						
gCad1						115.93				178.68		
gCad2										17.59		
GermD		85.59				247.29			537.79	1476.66		
Gua							56.05					
Hex				14.22								18.82
ICary										27.62		31.13
Isol										33.87		0.00
LaTerp						12.08						
LAlc		57.03		10.00				69.10			28.19	30.44
LAld								20.13			98.19	39.62
Led									491.93	202.01		
Ledo									28.26			
Lin		6.69				39.93						
Myris						381.50						

Compound Abbreviation	AUTB1	AUTC1	AUTG1	AUTH1	AUTI1	AUTK1	AUTL1	AUTN1	AUTO1	AUTQ1	AUTR1	AUTT1
Nace									35.77			
Non						6.34						
Palus									29.91			
SalAMe	30.71											
Spath1							30.97					
Sahex												
Terp												138.87
Thuje									13.56			
TaB1							49.00		123.15			
TaB2							16.86					96.33
TriCyc										452.22		
TriUnd												26.98
Zing						265.26	81.87		158.50			36.16
ZaBis												123.87
aTerpA						130.15				17.22		
bBis						112.48			149.65			154.38
bElem							148.16		67.95			44.55
EbFarn							80.71					
EbOci							14.19			78.29		
ZbOci							31.65					
bSes									43.73			
dTerp											39.82	26.57
dCad										87.05		
dEle									59.99			

Table S5. Tentative identification and estimated abundance (ng/g FW leaf bud) of compounds in New Zealand native resin samples collected in spring.

Compound Abbreviation	SPR E1	SPR D1	SPR H1	SPR K1	SPR M1	SPR I1	SPR J1	SPR N1	SPR O1	SPR Q1	SPR R1	SPR T1	SPR U1	SPR X1	SPR Y1	SPR Z1
aCub	9.12						50.84	28.84		29.17						
aPhel							18.39									
bMyrc	24.91								55.55	16.50						
yEle	23.98					53.64	178.17	22.15	21.01	113.17						
ZbFarn				15.22								16.56				
HDIC				9.19			36.34									
2Aceto				17.91												
2CampA									35.59	15.91						
2Cyc1					372.47											
ZHexAc		8.31														
Aden				15.50											1332.8	
aAmor				178.73						29.92						
aFarn												94.44				
aGurj										13.62						
aPin	432.40			10.08			205.82	2556.6	2339.6	420.56		184.61			68.99	
aCary	30.83						230.95			5.86		11.22				
AMC				17.01												
AVC	89.38										164.32		26.49		89.49	19.81
AmylC		21.80	14.54		8.53											51.81
bGurj							324.11	111.84								
bPin							17.92	22.12	125.11	33.41		30.63				
Cala							45.06									
Camp				15.19					72.96	170.60						
Camph										4.13						
Cary							18.29									

Compound Abbreviation	SPR E1	SPR D1	SPR H1	SPR K1	SPR M1	SPR I1	SPR J1	SPR N1	SPR O1	SPR Q1	SPR R1	SPR T1	SPR U1	SPR X1	SPR Y1	SPR Z1
CaryOx						24.57										
Cop	18.98								100.56	53.99						
Crith							96.09	341.33								
Cyc1M										4.93		22.61				
CycPen					18.29											
dLim	26.10								58.89	26.77						
Dnero						57.77										
Dum					17.84											
Ele						19.61										
Euc				32.06												
GermD										365.93						
Hex	8.40	10.23			8.84								8.33			37.17
ICary	68.34			24.37			125.05	19.39	57.27	22.50		39.73				
LaTerp												25.81				
lCamp				30.24												
LAlc					24.13		16.99				54.58					
LAld					19.32						9.68					5.41
Ledo						50.82		19.38								
Lin							203.81	51.45			12.06	6.61				
Myris				436.55												
Nero												12.02				
Nace							89.92									
Non		13.71														
pCim								20.35								
Sab	196.17						11.82		67.73	7.69						
Spath2						43.28	50.36									

Compound Abbreviation	SPR E1	SPR D1	SPR H1	SPR K1	SPR M1	SPR I1	SPR J1	SPR N1	SPR O1	SPR Q1	SPR R1	SPR T1	SPR U1	SPR X1	SPR Y1	SPR Z1
Sahex						8.61										
Terp				51.76												
Thuje								60.10		10.40						
TaB2												27.63				
TriCyc										572.29						
TriUnd											6.03					
VeFlor						96.36										
Zing				143.94								51.97				
ZaBis												125.14				
aCed												35.87				
aTerpA				135.27		29.82				7.80						
bBis				74.77								162.12				
bElem	19.91					45.52	748.14					172.05				
bFarn										70.84						
ZbOci							76.70			12.37						
dTerp				34.71		41.57										
dCad						137.95	108.22	64.37								

Table S6. Tentative identification and area under the peak for compounds in New Zealand propolis samples collected from beehives in autumn. AUT = Autumn, SOU = Southland, WAI = Waikato.

Sample	AUT SOU	AUT SOU	AUT SOU	AUT WAI	AUT WAI	AUT WAI	AUT WAI	AUT WAI	AUT WAI	AUT WAI	AUT WAI	AUT WAI	AUT WAI	AUT WAI	AUT WAI	AUT WAI
Replicate #	1	2	3	1	2	3	4	5	6	7	8	9	10	11	12	13
IsoA				11.3												
PreAce		4.65	3.38	6.95	9.21		11.26	1.55	4.49	7.67	4.22	11.29	13.16			20.17
Pre				0.66	1.43										5.07	
aMBA				2.83			2.8									
4PenAce				1.61												
aPin		3.15	0.82		18.88	6.22										
PryAce				2.27												
CevA				2.82			2.11									
BA				0.75				1.2								
Balc	4.12			15.77	0.81	3.21	6.28	10.44	11.96	5.26	12.75	12.94	2.98		4.99	
Lin		1.71	1.83	0.89	0.94											
PEAlc			0.94	4.4	0.8	3.35	3.4	3.29	2.57	3.33	3.35	3.85				
Phen				1.69				1.51								
BAce				4.34			0.62	0.83	2.18		1.05					
BCA				14.47												
bCyc			1.94	1.11			1.54									
ByAce				1.16												
TriOct				2.23	3.74											
TaB1		3.96	1.62	1.39	2.92	2.85			1.07	1.11			0.89			0.96
bHim				2.25	1.14											
aCurc				1.29	3.22	2.93										
aCop11	2.92			1.65	2.74	2.82				1.6	1.37	2.09	2.34	3.52		2.75
Camph					1.53											

Led					3.31											

Sample	AUT SOU	AUT SOU	AUT SOU	AUT WAI	AUT WAI	AUT WAI	AUT WAI	AUT WAI	AUT WAI	AUT WAI	AUT WAI	AUT WAI	AUT WAI	AUT WAI	AUT WAI	AUT WAI
bPin		1.6			1.53											
Lim			0.87		1.14	0.86						1.18				2.19
bFarn			1.29		1.3											
TaB2					6.38											
aCed		1.6				4.62										
Euc	3.34	2.43	1.41				1.85	1.36			0.83	2.4				
SAME							0.62									
IBBen			1.52				1.15									
aCub			0.72				1.4	1.44					0.93			
IPBen	3.16		3.87				1.47									
PB	11.01	11.18	4.53				5.86	1.94								
aMuur1							2.99	1.94			3.45	2.46	2.67	3.21		
GermD		1.21					3.16	2.77	1.75	2.94	1.34	3.4		4.32	2.94	
dCad		2.46	2.79				5.79	7.42	2.75	2.49	5.33	5.77	7.09	5.37	5.74	3.07
Gua							3.42	3.62	2.66	2.6	3.13					
Sulc								1.08	1.9		2.34					
dLim								1.49					1.39			
Aden								2.26			2.45	1.55	1.83	1.54		4.14
Cop		1.47	0.83					1.02			1.66	1.96	0.93	1.35		
Dec										0.47						
3Cyc1CA		1.77									1.33	1.77	0.98			
Val													5.24			
yCad														3.81		
Acep																1.93
Anis		1.91														

dNero		2.63	1.73													

Sample	AUT SOU	AUT SOU	AUT SOU	AUT WAI	AUT WAI	AUT WAI	AUT WAI	AUT WAI	AUT WAI	AUT WAI	AUT WAI	AUT WAI	AUT WAI	AUT WAI	AUT WAI	AUT WAI
ZbFarn	1.24	1.73														
AABE			1.53													
Nero			1.7													
aFarn			2.29													
3M3BB			13.11													
Cary													1.26			
Aroma						2.31										

Table S7. Tentative identification and area under the peak for compounds in propolis samples collected in spring from beehives in the upper half of the North Island of New Zealand. SPR = Spring, NOR = Northland, WAI = Waikato, HB = Hawke's Bay.

Sample	SPR NOR	SPR NOR	SPR NOR	SPR NOR	SPR NOR	SPR NOR	SPR NOR	SPR NOR	SPR NOR	SPR WAI	SPR WAI	SPR WAI	SPR HB	SPR HB	SPR HB	SPR HB	SPR HB	SPR HB	SPR HB	SPR HB	SPR HB	SPR HB	SPR HB	SPR HB	SPR HB	SPR HB
Rep.	1	2	3	4	5	6	7	8	9	1	2	3	1	2	3	4	5	6	7	8	9	10	11	12	13	14
PreAce		2.26	5.89	1.45	2.46	12.36	3.41	1.2	5.55	2.56	4.77	7.37	3.82	0.68		3.09		5.17		2.86	2.42	1.21	8.75	2.84	5.75	0.86
Pre			0.53				1.41	0.54				1.41						1.94			1.45		1.55		0.64	
aMBA																									1.36	
4PenAce																							1.08		19.4	
aPin		0.85	1.69	1.77		1.42	4.15	4.76	3.25						36.83	7.85	22.42	6.15	43.74	22.3		0.64	0.52	12.71	9.58	
PryAce												0.72											1.62			
Balc		3.18			1.56		1.31			1.68	13.26	1.82	2.44	2.26		3.65		2.51		1.44			2.81	1.5	0.59	0.64
Lin		4.52												2.67											0.35	
PEAlc		2.68	0.69	0.65	1.96	4.64	0.7		0.86	0.79	2.17		1.45	1.45		1.05		1.02				1.13	1.21	1.25		
Phen					1.31		1.86			1.96	4.57	2.22						1.29						0.36		
BAce		0.44									0.94															
bCyc		1.74		0.76																	0.98		0.81			
ByAce		0.71	1.67	1.88	2.1		2.09			2.29	4.97	1.69	0.32	1.45		1.89	2.07	2.66	0.29	1.49	1.64		3.92		1.32	0.59
TaB1		0.59	6.66	2.32	5.73		2.32	1.46	2.95	0.94		5.05	3.78	1.3		1.82	0	2.87			2.86	1.42	2.14	5.56	10.05	6.54
bHim					3.88		2.85																		1.01	
aCurc		1.58	5.87	3.65	2.56		1.92	1.88	3.11	3.23		8.33	1.05			2.1		5.22			6.17	1.3	4.44		13.98	8.05
aCop11		1.56	2.05	1.09	1.13				1.29	1.11		2.75	1.14					1.54			1.65	0.93	0.91		2.78	2.87
Camph															0.55				0.7							
bPin		1.38	3.54	3.27		2.42	2.29	3.33	5.48	1.21					44.1	11.61	39.85		39.34	33.27			0.94	1.56	0.32	
Lim									0.77								3.06			1.59						
bFarn			1.08		5.74																				0.95	

TaB2		0.94	2.2	0.48					1.34			1.75										0.42		1.11	1.69	1.45
aCed				1.7	0.65																					
Euc		4.26												16.28							5.09	0.3	2.31	1.42		

Sample	SPR NOR	SPR NOR	SPR NOR	SPR NOR	SPR NOR	SPR NOR	SPR NOR	SPR NOR	SPR NOR	SPR WAI	SPR WAI	SPR WAI	SPR HB	SPR HB	SPR HB	SPR HB	SPR HB	SPR HB	SPR HB	SPR HB	SPR HB	SPR HB	SPR HB	SPR HB	SPR HB	SPR HB
SAME										0.75												0.89		1.02		1.31
IBBen													1.54									2.37		1.7		3.65
aCub				0.85																						
IPBen				3.23								1.85										2.67				7.32
PB				7.07								3.79	7.34											13.72		
GermD		1.25																				0.7		2.11		
dCad		2.27		3.63	2.95								2.36			3						1.83		3.52		
Gua		9.44		5.65		3.69				1.97	1.86	0	1.91	7.72	1.16			2.62	0.26			8.75		2.1	0.65	
Sulc											1.3															
dLim															2.66				1.44							
Aden		1.97		1.8																				1.37		
Cop		0.86																	0.19			0.59		1.17		
Dec					0.83				1.14		0.55	0.4				0.41				1.22						
3Cyc1CA										1.22								0.65				0.73		1.12		0.75
Val		3.25																								
yCad				1.7																						
Acep																									0.65	
ZbFarn				0.51																						
dNero														0.54												4.06
Nero				3.34								2.43	2.28								2.81	7.04	2.42	2.93		
oMAP				0.46																						

Sample	SPR NOR	SPR NOR	SPR NOR	SPR NOR	SPR NOR	SPR NOR	SPR NOR	SPR NOR	SPR NOR	SPR WAI	SPR WAI	SPR WAI	SPR HB	SPR HB	SPR HB	SPR HB	SPR HB	SPR HB	SPR HB	SPR HB	SPR HB	SPR HB	SPR HB	SPR HB	SPR HB	SPR HB
aFarn				4.64																				0.8		1.91
bCych															0.64											
TaB1																										
Carv																										
BCyc		2.39																								
Thy	14.94																									
DiSty			2.29								2.43	2.04				2.48			0.25						0.53	
EbFarn			0.62	0.25	7.17							1.39				1.2		0.53			1.16					
Sulc			1.64							0.95												0.31				
Non						9.4														1.07						0.64
BAPy																						0.93				1.56
Enero																									0.55	
DiBi			4.42																						6.12	
Zing				0.52	2.63							0.98				1.86									2.78	
aBis							3.18					1.8														
bSes																									1.3	
bBis			0.53																						1.31	
5Az										2.04																
ZbOci															4.07				5.26	2.21						
3M3BB																										1.57
Sab															0.3											
EbOci																	7.9									
LaTerp				0.63										1.22	0.84					1.6						
LtrPino															1.45	1.41			0.44	1.05						
Myrta															1.21				0.47							

Hemim			1.42	0.7																						
Spath									7.4																	
BCycBu													0.81													
IsoE													1.4												1.42	
Sahex																			0.1							
tertB															0.42											

Sample	SPR NOR	SPR NOR	SPR NOR	SPR NOR	SPR NOR	SPR NOR	SPR NOR	SPR NOR	SPR NOR	SPR WAI	SPR WAI	SPR WAI	SPR HB	SPR HB	SPR HB	SPR HB	SPR HB	SPR HB	SPR HB	SPR HB	SPR HB	SPR HB	SPR HB	SPR HB	SPR HB	SPR HB
aCamp															0.52											
Nopi															0.57											
Sabi															1.12				0.37							
Cycpro																1.26										
aCary		1.1								3.71								7.35		4.39	7.99		10.56	2.2		2.25
Wid																						1.98				
3Car																			0.16							
7Endo																			0.31							
bPhel																			0.31							
PinoC																			0.23							
nBIso																							0.55			
PA2Met																							5.74			
PACI					10.8			5.73				4.85												8.02		3.64
Cary		3.64								2.79		1.05		2.64				5.82		4.2	5.33		8.48	1.85		
aGua		2.25																								
Aroma		1.74																								
Cychept		1.88																								
TetCyc							15.14																			

Cala	7.05	7.8
		28.0
Laur	7.72	1

Table S8. Tentative identification and area under the peak for compounds in propolis samples collected in spring from beehives in the lower half of the North Island of New Zealand. SPR = Spring, GIS = Gisborne, MAN = Manawatu-Whanganui.

Sample	SPR GIS	SPR GIS	SPR GIS	SPR GIS	SPR MAN	SPR MAN	SPR MAN	SPR MAN
Replicate	1	2	3	4	1	2	3	4
PreAce		2.23		3.82			1.47	
Pre	3.37							
BA					1.78			
Balc		3.18		12.84	14.44			3.3
Lin		0.37						
PEAlc		1.46		2.22				1.57
BAce				1.9				
ByAce	3.26	3.25	1.33	1.7	1.98	4.73		
TaB1	1.29	1.67	3				2.66	
bHim			5.26	1.28				
aCurc	9.14		4.63					
aCop11	2	0.47	1.24					
bPin								3.07
TaB2	9.45	4.75	0.91					
Euc		3.42						
IBBen				1.48				
IPBen				3.56				
PB				4.13	5.24		5.24	
aMuur1					1.05			
dCad					1.76			
Gua		1.66		1.41	3.33			
Sulc				0.66				
3Cyc1CA							2.03	

Sample	SPR GIS	SPR GIS	SPR GIS	SPR GIS	SPR MAN	SPR MAN	SPR MAN	SPR MAN
AABE		2.14						
Nero					1.46		1.85	
oMAP					2.99			
aFarn	1.67	2.24	1.61		2.05			
bCych						4.82		
CycTet						2.62		
Carv			3.47	0.82				
Bcyc								3.85
Thy	18.11	12.75	14.83	29.09			7.64	
DiSty	1.87	3.2	2.94	1.71				
EbFarn	1.65							
ZEaFarn	2.45							
Non			2.6					
lCamp				0.56				
Hthy				1.36				
BAPy				0.8				
Enero				1.43				
IsoE								2.67
nBIso					2.89			

Table S9. Tentative identification and area under the peak for compounds in propolis samples collected in spring from beehives in the South Island of New Zealand. SPR = Spring, NEL = Nelson, NCAN = North Canterbury, CAN = Canterbury, SCAN = South Canterbury, WC= West Coast, SOU = Southland.

Sample	SPR NEL	SPR NEL	SPR NEL	SPR NCAN	SPR NCAN	SPR NCAN	SPR NCAN	SPR CAN	SPR CAN	SPR CAN	SPR CAN	SPR SCAN	SPR SCAN	SPR SCAN	SPR WC	SPR WC	SPR WC	SPR WC	SPR WC	SPR SOU	SPR SOU	SPR SOU
Replicate #	1	2	3	1	2	3	4	1	2	3	4	1	2	3	1	2	4	3	5	1	2	3
PreAce		2.96	1.32		0.4	1.2	0.23	1.61			3.58	5.52				1.09	6.75	5.4			3.31	0.44
Pre									1.59									0.9				
aMBA																						0.65
4PenAce																	0.61	0.52				
aPin		1.93		57.01	11.23								11.76	9.26	34.48	25.15			0.81	4.23	2.21	
BA								3.84														
Balc			2.27	1.74				11.52											7.81			
Lin											0.46							0.78		2.47	1.87	1.24
PEAlc			1.18	0	1.05						0.96								1.06			
Phen											1.41	1.12										3.33
BAce								2.56											0.75			
bCyc														1.47					0.47			
ByAce								1.71	2.25	2.94	2.57						0.55					0.69
TaB1	2.61	14.44	6.29		0.37	1.23	1.14		6.28	3.46	8.9	5.25	7.68			1.32	4.49	13.2	4.63	11.19	0.45	8.33
bHim													3.97					10.07				
aCurc	5.67	15.78	9.73		0.72	1.69	1.53			2.66	19.95	12.13	6.49			2.66	4.95	10.02	4.06		0.21	5.62
aCop11	1.75	1.92	2.68	0.7			0.54		2.4	2.58	2.14	2.43	2.2			1.37	5.11	5.43			1.28	
Camph															0.83				2.66			
bPin	2.91			0.73	0.3							0.9	12.99	1.08	39.49	22.9			1.11			
bFarn																					2.3	9.7
TaB2		4.7	1.57		0.76				1.52	1.41	2.02	1.23	2.3			1.6	3.63	3.33	1.81			3.17
aCed										2.03	2.38							2.55		4.29	2.79	5.24
Euc										1.63	0.81	3.53								4.22	1.82	

Sample	SPR NEL	SPR NEL	SPR NEL	SPR NCAN	SPR NCAN	SPR NCAN	SPR NCAN	SPR CAN	SPR CAN	SPR CAN	SPR CAN	SPR SCAN	SPR SCAN	SPR SCAN	SPR WC	SPR WC	SPR WC	SPR WC	SPR WC	SPR SOU	SPR SOU	SPR SOU
SAME																				3.44		
IBBen														6.16						2.32	2.85	
aCub																						2.01
IPBen														11.31	1.39						6.63	
PB														7.21	0.82						16.13	
aMuur1														0.82								
GermD					1.51	0.87																
dCad					2.39	1.3	2.06							1.88	0.32						3.75	2.32
Gua	8.45						0.36			5.39		0.14									0.76	5.62
Sulc								6.14			1.1											
dLim				1.64											2.23							
Aden							0.64														2.07	1.05
Cop					0.52		0.58							0.73						1.29	1.71	0.92
Dec												0.84					0.35					0.44
3Cyc1CA																				2.85	1.79	1.54
yCad							2.16														1.63	
Acep												0.9					0.53	0.9				
ZbFarn			0.84				0.74						0.65					1.93		7.23		
dNero	2.65						1.01								0.75	3.26				4.5		
Nero				1.14	0	0.97				2.65		8.74		3.52							4.17	
oMAP	2.38		1.99																			
aFarn			2.56							0.93	2.41						0.97				4.17	
TaB1																				3.53	1.98	
DiSty									1.37		1						1.03					
EbFarn		1.36							1.5	1.68							0.54	0.51		7.03		5.62

Sample	SPR NEL	SPR NEL	SPR NEL	SPR NCAN	SPR NCAN	SPR NCAN	SPR NCAN	SPR CAN	SPR CAN	SPR CAN	SPR CAN	SPR SCAN	SPR SCAN	SPR SCAN	SPR WC	SPR WC	SPR WC	SPR WC	SPR WC	SPR SOU	SPR SOU	SPR SOU
EbFarn		1.36							1.5	1.68							0.54	0.51		7.03		5.62
ZEaFarn		3.02									0.68											
Non		0						5.79							0.32	0.52		1.77	1.35	1.41		
Hex								1.68														0.39
DiBi		11.89	5.33	0.45					3.09		5.43		6.99				7.23	11.61	1.78			
Zing							0.5			2.89							5.24	3.87		3.9	1	3.54
aBis										4.05									2.6			
bSes		1.36																1.96				2.36
GermB		7.83																		2.77		
bBis		2.44																0.56				
ZbOci				0.62										0.97		2.53					1.67	
BAPe														2.64								
3M3BB														1.6	3.03						2	
bCych				0.6											0.82							
oCy															0.53							
Sab															0.62					4.63		
EbOci															3.8							
b-Pino															0.59							
trPino															1.19							
ScisV															0.92							
tr3Pin															0.56							
LaTerp															0.65							
Myrto															1.24							
LtrPino																1.31						
Myrta																0.85						
VA					0.23														1.59			

Sample	SPR NEL	SPR NEL	SPR NEL	SPR NCAN	SPR NCAN	SPR NCAN	SPR NCAN	SPR CAN	SPR CAN	SPR CAN	SPR CAN	SPR SCAN	SPR SCAN	SPR SCAN	SPR WC	SPR WC	SPR WC	SPR WC	SPR WC	SPR SOU	SPR SOU	SPR SOU
PPr																			3.12			
Epig																			15.78			
Ledo																			5.28			
3CIco				0.52			1.47															
Hemim				0.51	0.99		0.85															
2Med				0.36																		
4Terp				0.72																		
TME				0.79																		
Cume					0.23																	
trDec					0.61		0.96															
tr4Car					0.44																	
CychexP					0.5																	
aMuur2					0.99																	
Spath					2.15	1.14																
2M1Oc						2.58																
BCycB				0.9			0.86															
Ced							1.38															
BCycBu																						
7NBB																					1.49	
PACI																						4.61
Cary																						2.59
Aroma					0.79																	
Sati																						2.11
CapA																						0.79
